# Availability and experiences of differentiated antiretroviral therapy delivery at HIV care facilities in rural Zimbabwe: a mixed‐method study

**DOI:** 10.1002/jia2.25944

**Published:** 2022-08-25

**Authors:** Benedikt Christ, Janneke H. van Dijk, Talent Y. Nyandoro, Martina L. Reichmuth, Cordelia Kunzekwenyika, Frédérique Chammartin, Matthias Egger, Alison Wringe, Marie Ballif, for the International epidemiology Databases to Evaluate AIDS in Southern Africa (IeDEA‐SA)

**Affiliations:** ^1^ Institute of Social and Preventive Medicine University of Bern Bern Switzerland; ^2^ SolidarMed Masvingo Zimbabwe; ^3^ Centre for Infectious Disease Research and Epidemiology University of Cape Town Cape Town South Africa; ^4^ Population Health Sciences Bristol Medical School University of Bristol Bristol UK; ^5^ London School of Hygiene and Tropical Medicine London UK

**Keywords:** HIV, ART, differentiated care, differentiated ART delivery, Africa, Zimbabwe

## Abstract

**Introduction:**

Zimbabwe adopted differentiated HIV care policies in 2015 to promote client‐centred care and relieve strain on health facilities. We examined the availability, experiences and perceptions of differentiated antiretroviral therapy (ART) delivery in rural Zimbabwe following the policy adoption.

**Methods:**

We undertook a cross‐sectional mixed methods study in all the 26 facilities providing HIV care in a rural district in Zimbabwe. We collected quantitative data about ART delivery and visit durations from 31 healthcare providers and a purposive stratified sample of 378 clients obtaining ART either through routine care or differentiated ART delivery models. We performed 26 semi‐structured interviews among healthcare providers and seven focus group discussions (FGDs) among clients to elicit their perceptions and experiences of ART delivery. Data were collected in 2019, with one follow‐up FGD in 2021. We analysed the transcripts thematically, with inductive coding, to identify emerging themes.

**Results:**

Twenty facilities (77%) offered at least one differentiated ART delivery models, including community ART refill groups (CARGs; 13 facilities, 50%), fast‐track refill (8, 31%), family refill (6, 23%) or club refill (1, 4%). Thirteen facilities (50%) offered only one model. The median visit duration was 28 minutes (interquartile range [IQR]: 16–62). Participants in fast‐track had the shortest visit durations (18 minutes, IQR: 11–24). Confidentiality and disclosure of HIV status, travelling long distances, travel costs and waiting times were the main issues influencing clients’ views on differentiated ART delivery. Fast‐track refill was perceived as the preferred model of clients for its limited involuntary disclosure and efficiency. In contrast, group‐ and community‐based refill models reduced travel costs but were felt to be associated with involuntary disclosure of HIV status, which could discourage clients. Healthcare providers also experienced an additional workload when offering facility‐based group models, such as CARGs.

**Conclusions:**

Differentiated ART delivery models were widely available in this rural setting, but most facilities did not offer a choice of models to address clients’ diverse preferences. A minority offered fast‐track refills, although this model was often mentioned as desirable. Confidentiality, travel expenses and client waiting times are key elements to consider when planning and rolling out differentiated HIV care.

## 1 INTRODUCTION

As the number of people living with HIV (PLHIV) on life‐long antiretroviral therapy (ART) increases, maintaining long‐term engagement in care without overburdening health systems is a challenge, especially in sub‐Saharan Africa. In 2019, an estimated 25.4 million PLHIV were receiving ART globally, and among them, 17.9 million in sub‐Saharan Africa [1]. The traditional “one‐size‐fits‐all” model of care, whereby PLHIV have regular individual clinic visits, stretches health facilities to their limits and does not always meet clients’ diverse needs and their preference for tailored services [2, 3, 4].

Differentiated service delivery (DSD) offers client‐centred HIV services across the cascade of care, from HIV testing to ART refills, and was endorsed by the World Health Organization in 2016 [5, 6, 7]. Community‐ and facility‐based differentiated ART delivery models can play an important role in decongesting HIV care facilities by reducing the frequency of clinic visits and shortening their duration [8, 9]. Furthermore, differentiated ART delivery has the advantage of improving adherence to ART and retention in care, although this may vary by model [10, 11, 12, 13]. However, implementation barriers, including insufficient staff training, lack of dedicated personnel or insufficient support and resources, may prevent the successful implementation and uptake of differentiated ART delivery [14, 15, 16]. Health emergencies, such as the recent coronavirus (COVID‐19) pandemic, can also challenge the HIV care delivery, especially where services are already stretched [17, 18]. In this context, DSD can reduce the burden on providers by offering community‐ and facility‐based models of ART delivery with fewer clinic visits [19].

Zimbabwe carries a heavy burden of HIV, with an estimated HIV prevalence among adults of 12.8% and 1.1 million people on ART in 2019 [1]. The Zimbabwean Ministry of Health and Child Care (MoHCC) adopted and rolled out DSD in 2015. The MoHCC guidelines published in 2017 recommend implementing differentiated ART delivery at all HIV care facilities in the country and provide a decision framework to support the identification of ART delivery models that should be offered based on the available resources and the specific needs of the clients in care at each facility [20]. However, little is known about the availability of differentiated ART delivery models in rural areas or the perceptions and experiences thereof among clients and healthcare providers. In this mixed‐method study, we combined quantitative and qualitative methods to determine the availability of differentiated ART delivery models, compared facility‐based visit durations across models and explored the perceptions and experiences of clients and healthcare providers in rural Zimbabwe.

## 2 METHODS

### 2.1 Study design and setting

**Figure 1:**
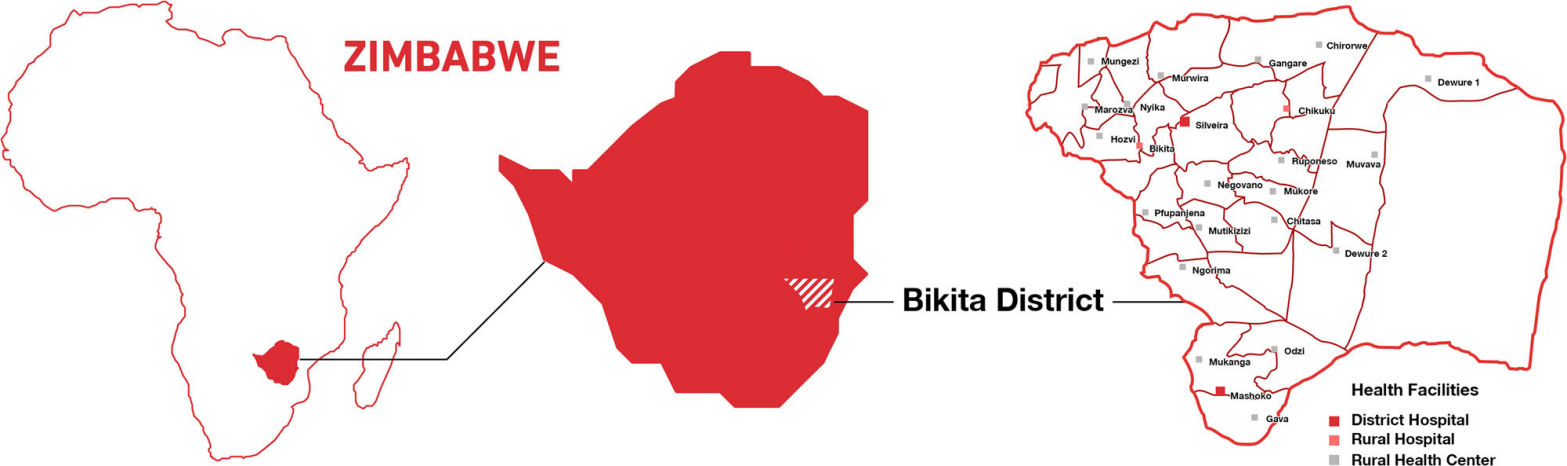
Map of Bikita District in Zimbabwe, including the 26 participating health facilities.

We conducted a cross‐sectional study in all the 26 HIV care facilities available in Bikita District, Province of Masvingo, Zimbabwe, in 2019 (Figure 1). All 26 facilities were in rural settings. Bikita District had a population of 174,068 at the last census in 2012, in an area covering 5,180 km^2^, and most residents are subsistence farmers. HIV care at all facilities was supported by SolidarMed, a non‐governmental health organization working in partnership with the Zimbabwean MoHCC. Eighteen facilities were members of the International Epidemiology Databases to Evaluate AIDS in Southern Africa (IeDEA‐SA) consortium [21]. The study included a questionnaire‐based survey among PLHIV and healthcare providers, semi‐structured interviews with healthcare providers and focus group discussions (FGDs) with PLHIV. Data collection tools were adapted from questionnaires provided in the national DSD manual [20]. We collected survey and interview data at each facility during 2 consecutive days and conducted FGDs at selected facilities on separate days, between May and December 2019. We ran one additional FGD in March 2021 to garner further insights into the perspective of clients enrolled in the fast‐track model.

### 2.2 Definitions

**Figure 2:**
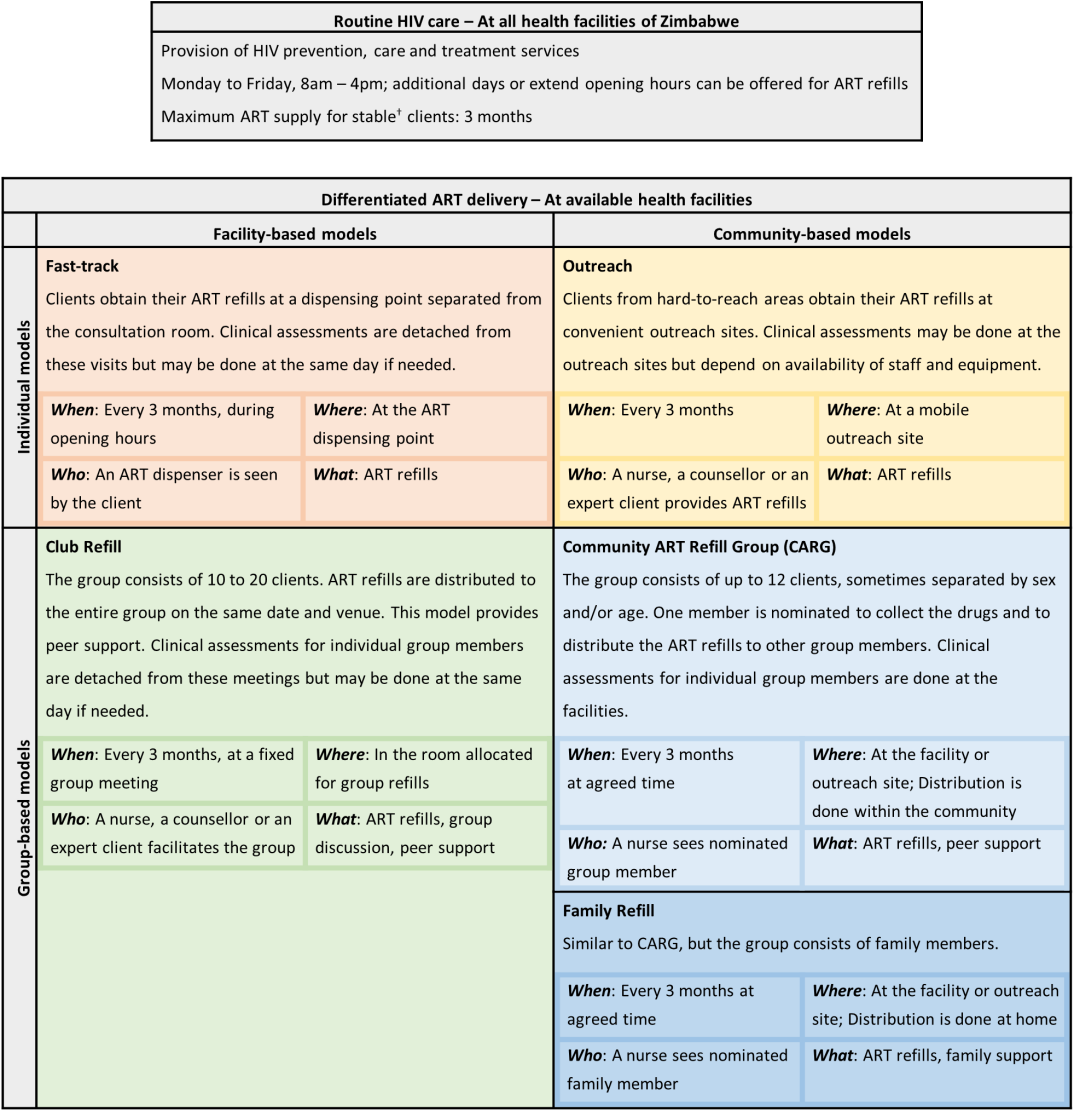
Description of routine HIV care and the five differentiated ART delivery models described by the national DSD manual of Zimbabwe. Adapted from [20, 43]. ^†^ Clients are considered as stable and are eligible for differentiated ART delivery if they have no opportunistic infections, are at least six months on ART, and have a viral load below 1,000 copies/ml, or a CD4 cell count above 200 cells/mm^3^ if viral load testing is unavailable. Abbreviations: ART, Antiretroviral therapy; CARG, Community ART Refill Group.

Three levels of facilities were included in this study. Rural health centres provided primary outpatient care and were staffed by a small team of nurses. Rural hospitals offered basic in‐patient and laboratory services. District hospitals were the largest rural facilities and offered comprehensive in‐patient care. Routine HIV care was available at all facilities for all PLHIV. Clients in routine care were offered ART refills for a maximum of 3 months; visits included ART refills and clinical reviews. Aside from routine HIV care, Zimbabwe adopted five models of differentiated ART delivery [20]: two facility‐based (fast‐track and club refill) and three community‐based models (mobile outreach, community ART refill group [CARG] and family refill). The models differ by location, provider and whether they are for individual clients or groups (Figure 2). Clients stable on ART (no opportunistic infections, at least 6 months on ART, viral load below 1,000 copies/ml or a CD4 cell count above 200 cells/mm^3^) were eligible for differentiated ART delivery, including pregnant/breastfeeding women, children and young people as per MoHCC guidelines [20]. The clinic visit duration was defined from the time a client arrived at the facility until departure.

### 2.3 Quantitative data collection and analyses

At each facility, we invited one consenting HIV healthcare provider who had worked for at least 1 year at the facility to participate in the study. Since most facilities were small, there was often only one provider on duty. If there were more, we selected the provider most experienced in HIV care. Questions included facility characteristics, available differentiated ART delivery models and perception of workload. In addition, we recruited a purposive stratified sample of PLHIV on ART for at least 6 months, including caregivers of children aged 0–15 years, young people aged 16–24 years and adults aged over 24 years. Clients were eligible regardless of the mode of ART refill, that is either through routine care or any of the available differentiated ART delivery models. The study team approached eligible PLHIV attending the facility at their arrival, explained the study and asked them if they were willing to participate. All participants in the study provided informed consent. Data obtained from PLHIV included demographics and their model of ART delivery. We collected arrival and departure times using a stamp‐card in a sub‐sample of participants. Participants received the stamp‐card upon arrival at the facility and returned it to the study team when they left, and nurses indicated the corresponding arrival and departure times on the card. We collected the data on Android electronic devices using REDCap (www.projectredcap.org) [22, 23]. We used descriptive statistics to summarize the availability of differentiated ART delivery models at facilities and describe the clinic visit durations by the ART delivery model. Analyses were performed with R v3.6 [24].

### 2.4 Qualitative data collection and analyses

We invited the participating HIV healthcare providers to individual semi‐structured interviews. If available, a second healthcare provider participated (paired interviews). Interview guides included topics on perceived healthcare providers’ experiences in delivering HIV care, and clients’ barriers and challenges in accessing ART. We also performed seven FGDs of 6–10 PLHIV on ART for at least 6 months, each at different facilities. Participants were purposively selected to cover different age, gender and key population categories, at facilities offering at least one differentiated ART delivery model, and where at least six clients consented to participate. We invited clients enrolled in one of the differentiated ART delivery models, or in routine care, to participate in the FGDs and share their experiences or perceptions of differentiated ART delivery. The topic guide covered questions on perceived barriers and facilitating factors in accessing ART and using differentiated ART delivery models.

Semi‐structured interviews lasted 30–60 minutes and were performed by two trained interviewers. FGDs lasted 60–120 minutes and were conducted by a trained facilitator and a note‐taker, matched by gender to the participants. In the case of mixed genders within the FGD, we ensured that the team of facilitators was also mixed. We conducted interviews and FGDs at the facilities ensuring participants’ privacy and comfort. Interviews and FGDs were done in Shona, audio‐recorded, transcribed and translated into English. We inductively coded the data to identify patterns and uniformities in the data related to the research objectives. We used a thematic analysis to identify codes emerging from the data. We then grouped codes into categories, narrowing them down to themes, which we continued to constrict until no further themes emerged [25]. We used Atlas.Ti (Version 8.4.4) to code and manage the transcripts.

### 2.5 Ethics

The Cantonal Ethics Committee Bern (Switzerland) and Provincial Medical Directorate of the Ministry of Health and Child Care (Zimbabwe) approved this study.

## 3 RESULTS

### 3.1 Characteristics of participating sites and availability of differentiated ART delivery models

Among the 26 facilities in the district, 22 (84%) were rural health centres, two (8%) were rural hospitals and two (8%) were district hospitals (Figure 1 and Table 1). The facilities comprised a median of 389 clients in care (interquartile range [IQR]: 211–688), ranging from 6 to 1,271.

**Table 1:** Characteristics of the 26 participating facilities and availability of differentiated ART delivery models

	Total	Rural health centre	Rural hospital	District hospital
	26 (100%)	22 (84%)	2 (8%)	2 (8%)
Facility owner				
Council	12 (46%)	12 (55%)	–	–
Government	10 (38%)	8 (36%)	2 (100%)	–
Mission	3 (12%)	1 (5%)	–	2 (100%)
Private	1 (4%)	1 (5%)	–	–
Median number of clients in care (IQR)	389 (211–688)	346 (180–433)	994 (881–1,106)	1,152 (1,146–1,157)
Median number of HIV care providers (IQR)	4 (3–5)	4 (3–4)	7 (6–8)	12 (10–14)
Median number of differentiated ART delivery models aside from routine HIV care (range)	1 (0–3)	1 (0–2)	2 (0–3)	2 (1–2)
Available differentiated ART delivery model^a^				
CARG	13 (50%)	11 (50%)	1 (50%)	1 (50%)
Club refill	1 (4%)	–	–	1 (50%)
Family refill	6 (23%)	5 (23%)	1 (50%)	–
Fast‐track	8 (31%)	6 (27%)	1 (50%)	1 (50%)
Outreach	–	–	–	–

Abbreviations: ART, antiretroviral therapy; CARG, community ART refill group; IQR, interquartile range.

^a^ At some facilities, more than one differentiated ART delivery model were available.

The facilities offered routine HIV care and up to three differentiated ART delivery models: 13 (50%) had one, six (23%) had two and one (4%) had three models. In total, 13 facilities (50%) offered CARG, eight (31%) fast‐track, six (23%) family refill and one facility (4%) offered club refill. Six (23%) facilities provided only routine HIV care, and outreach was not available at any facility (Table 1).

### 3.2 Characteristics of participating PLHIV and enrolment in differentiated ART delivery models

We collected data from 378 PLHIV at 25 facilities; one of the rural health centres did not have HIV consultations on the day of data collection. A median of 16 PLHIV participated at each facility (IQR: 12–19). The median age of participants was 41 years (IQR: 32–51 years); 245 (65%) were female. The majority of participants (303, 80%) received ART through routine HIV care, 32 (8%) were enrolled in fast‐track, 30 (8%) in CARGs, seven (2%) in a family refill and six (2%) in club refill model. Further characteristics were available for 313/378 (83%) participants. The median time on ART was 6 years (IQR: 4–9 years), participants had a median of four clinic visits per year (clinical consultations and/or ART refills) and their median travel time from home to the facility was 60 minutes (IQR: 30–120). We obtained the clinic visit duration for 204/378 (54%) participants. The median visit duration was 28 minutes (IQR: 16–62), from arrival at the facility until departure, the shortest being for participants on fast‐track (18 minutes, IQR: 11–24), the longest for family refill members (91 minutes, IQR: 72–106; Table 2).

**Table 2:** Characteristics of PLHIV participating in the quantitative survey and enrolment in differentiated ART delivery models

		ART delivery model				
	Overall 378 (100%)	Routine HIV care 303 (80%)	CARG 30 (8%)	Club refill 6 (2%)	Family refill 7 (2%)	Fast‐track 32 (8%)
Gender						
Female	245 (65%)	196 (65%)	20 (67%)	6 (100%)	4 (57%)	19 (59%)
Male	133 (35%)	107 (35%)	10 (33%)	0	3 (43%)	13 (41%)
Median age in years (IQR)	41 (32–51)	40 (31–50)	48 (35–55)	56 (43–68)	45 (38–59)	42 (32–52)
Level of care						
District hospital	36 (9%)	27 (9%)	3 (10%)	6 (100%)	–	–
Rural hospital	45 (12%)	24 (8%)	7 (23%)	–	–	14 (44%)
Rural health centre	297 (79%)	252 (83%)	20 (67%)	–	7 (100%)	18 (56%)
Median time on ART in years (IQR)^a^	6 (4–9)	6 (3–8)	8 (7–10)	7 (4–9)	4 (3–5)	6 (4–8)
Median number of clinic visits per year (IQR)^a^	4 (4–4)	4 (4–4)	4 (4–12)	5 (4–8)	4 (4–4)	4 (4–4)
Median travel time from home to the facility in minutes (IQR)^a^	60 (30–120)	60 (30–120)	45 (30–120)	90 (82–98)	60 (60–105)	52 (30–82)
Median visit duration, from arrival until departure in minutes (IQR)^b^	28 (16–62)	32 (16–62)	22 (16–138)	28 (24–30)	91 (72–106)	18 (11–24)
Median number of health services received during the visit^c^ (IQR)^b^	2 (2–3)	2 (1–3)	2 (2–3)	3 (2–4)	3 (3–4)	3 (3–4)

Abbreviations: ART, antiretroviral therapy; CARG, community ART refill group; IQR, interquartile range; PLHIV, people living with HIV.

^a^ Available for 313 participants.

^b^ Available for 204 participants.

^c^ Health services, such as blood test, counselling, clinical consultation and ART refill.

### 3.3 Factors influencing the preference for differentiated ART delivery models

The characteristics of PLHIV and healthcare providers who participated in FGDs and interviews are listed in Tables 3 and 4, respectively. Three themes emerged regarding the issues that influenced preferences and perspectives of different differentiated ART models among PLHIV and healthcare providers: (1) confidentiality and disclosure, (2) travelling long distances and associated expenses, and (3) waiting times.

**Table 3:** Characteristics of the 57 PLHIV participating in the seven focus group discussions and description of facilities where discussions took place

	Children^a^ *Routine care and CARG*	Young people *Routine care*	Young people *Club refill*	Pregnant or breastfeeding women *Routine care*	Non‐pregnant or breastfeeding women *Routine care and CARG*	Adult men *Routine care*	Adult women and men *Fast‐track* ^b^
Number of participants	7	8	10	9	7	6	10
Gender	3 girls	4 women	7 women	9 women	7 women	6 men	9 women
	4 boys	4 men	3 men				1 man
Median age in years (IQR)	8 (8–9)	14 (12–16)	18 (17–19)	34 (26–41)	50 (43–52)	49 (45–63)	50 (40–58)
Mode of ART delivery	4 on routine HIV care	8 on routine HIV care	10 in club refill	9 on routine HIV care	4 on routine HIV care	6 on routine HIV care	10 in fast‐track
	3 in CARGs				3 in CARGs		
Type of the attended facility	Rural health centre	Rural health centre	District hospital	Rural health centre	Rural health centre	District hospital	Rural health centre
Date of focus group discussions	September 2019	September 2019	December 2019	August 2019	August 2019	September 2019	March 2021
Available differentiated ART delivery model(‐s) at the facility aside from routine HIV care (year of model introduction)	CARG (2019)	Fast‐track (2018)	Club refill (2018)	Fast‐track (2018)	CARG (2018)	Fast‐track (2018)	Fast‐track (2020)
		CARG (2018)	CARG (2018)	Family refill (2018)			CARG (2018)

Abbreviations: ART, antiretroviral therapy; CARG, community ART refill group; IQR, interquartile range; PLHIV, people living with HIV.

^a^ Children were represented by their caregivers.

^b^ Additional focus group discussion conducted in March 2021 to gain insights into the perspectives of clients enrolled in the fast‐track model.

**Table 4:** Characteristics of the 31 healthcare providers participating in the face‐to‐face interviews

	Characteristics
Median age in years (IQR)	39 (35–47)
Gender	21 women
	10 men
Profession	31 nurses
Median years working at the facility (IQR)	3 (2–12)
Interview type	21 individual interviews
	5 paired interviews

Abbreviation: IQR, interquartile range.

### 3.4 Confidentiality and disclosure

Healthcare providers and clients indicated that a major barrier to the uptake of differentiated ART delivery models resided in clients’ concerns around confidentiality and unwanted disclosure of their HIV status: “Everything comes back to issues of confidentiality.” (Young woman on routine HIV care, rural health centre). For clients who had not disclosed their HIV status and were concerned about maintaining confidentiality, fast‐track was often identified as the preferred choice of ART delivery model: “You will not be seen by others who are not on ART.” (Woman in fast‐track, rural health centre). This was confirmed by healthcare providers: “Fast‐track will be more appropriate […]. We have uniformed forces, teachers and others who would want to always maintain confidentiality.” (Healthcare provider, rural hospital). However, being seen at the facility can also be a source of fear: “Coming to the health facility, like today is my medication collection day, I have fear when I see someone I know […].” (Woman in fast‐track, rural health centre). In contrast to fast‐track, group‐based refill models, such as CARGs, family and club refills, were felt to be associated with involuntary disclosure and clients perceived them as problematic:

With HIV, most of the people do not want to be known that their children are on [antiretroviral therapy]. So, joining a group of people (CARGs or club refills) will be problematic in the sense that some other people cannot keep confidential issues, so the whole village will end up knowing. (Caregiver of a child on routine HIV care, rural health centre)

This was reflected on by clients enrolled in group‐based models who were afraid of being exposed: “We do not want to be seen taking medication […] as we fear of being ridiculed by peers” (Young woman in club refill, district hospital). Similar observations were also made by healthcare providers, who recognized the advantage of fast‐track compared to group‐based models to protect privacy:

The model that we are using is fast‐track, as for other models like CARGs, looking at our community, that will not be viable, because they will now be knowing that this one is on ART, and the word spreads and that's the thing that they don't want to be known. (Healthcare provider, rural health centre)

Clients enrolled in the fast‐track model confirmed that privacy and confidentiality were preserved at the facilities: “When we come, we just wait in the queue like [other outpatient] patients [with negative HIV status], but others go straight to the pharmacy, as for me I just act like an [outpatient] patient.” (Woman in fast‐track, rural health centre).

For some clients, trust and confidentiality between family members were also considered as sensitive: “When there are disputes within the family, they will be the ones to disclose your status to the community without your consent.” (Man on routine HIV care, district hospital).

For these clients, family refill would not be suitable to preserve their privacy. Furthermore, expulsion and stigma could also arise within families:

Sometimes people discriminate against us because they are not in it [in a differentiated ART model]; some of our relatives when we have conflicts, they will disclose your [HIV] status […]. We really encounter this with some of our relatives sometimes. (Woman in fast‐track, rural health centre)

The outreach model was not available at any facility; yet, even this individualized community‐based model was perceived as insufficient to meet the confidentiality needs of clients, as it would draw attention to the outreach vehicle:

“It is not good, because when the medication vehicle comes in the community, everyone will want to know what it is for.” (Man in fast‐track, rural health centre). It was also anticipated that clients on ART going to pick up their refills at outreach sites would be demarcated: “The disadvantage is that people will start saying ‘the vehicle for those on ART has come’.” (Woman in fast‐track, rural health centre).

Confidentiality and disclosure issues eventually impaired the implementation of CARGs at some facilities: “CARGs, we have failed to implement due to issues of disclosure within the patients themselves.” (Healthcare provider, rural health centre).

### 3.5 Travel and associated expenses

Many clients highlighted challenges with securing transport money to access facilities. A lack of transport money often results in clients walking long distances, which in some instances could affect the utilization of healthcare services: “We face challenges of transport money.” (Woman on routine HIV care, rural health centre). Healthcare providers also confirmed this: “Many patients come from far away and transport cost is usually a problem for them.” (Healthcare provider, rural hospital).

Group‐based refill models, including CARGs, can have the advantage to reduce the number of times clients come to the facilities and hence the challenge of travelling long distances: “CARGs will [address] issues of distances [to the facility], which for some of us are a challenge.” (Caregiver of a child on routine HIV care, rural health centre). Group‐based refill models also relieved clients and caregivers of children who were unable to walk long distances, and allowed them to care for their responsibilities at home, while others collected the drugs for them: “It helps the elderly [clients] because in CARGs, the elderly do not come to the clinic, but other members collect [ART] for them.” (Woman in CARG, rural health centre). Healthcare providers confirmed that CARGs were the best ART delivery options to address the challenges of long walking distances and travel expenses:

CARG may best address our challenges, since we noticed that individuals often face the challenge of obtaining fares for transportation as they often travel long distances. It's harder for them as individuals. As such CARG has an advantage in that, when they take turns, a person may have ample time to save money until their turn comes. (Healthcare provider, rural health centre)

Outreach models could address the issues of long distances to the facilities and travel costs but were not available in the district. However, clients considered a clear advantage of the outreach model in reducing the distances to the facilities: “The issue of walking would be addressed since others have problems with legs” (Woman in fast‐track, rural health centre). However, the implementation of the outreach model was challenged by costs, as there were no vehicles to deliver the drugs to the designated points, no funds for fuel and staff allowances to pay: “We don't have the necessary support in implementing the other models, for example, the outreach cannot be implemented since we have no transport vehicle.” (Healthcare provider, rural health centre).

Aside from differentiated ART delivery models, 6‐month drug supply instead of 3‐month supply was welcomed by clients: “They are also being good to us because there is now a six‐month supply […]” (Woman in fast‐track, rural health centre). The shift from 3‐ to 6‐month drug supply could help to reduce the frequency of refill visits and thus save transport money.

### 3.6 Waiting time

Clients expressed their concern about long waiting times, which rendered clinic visits hard to integrate in their daily life: “The long waiting time is a challenge because we will be hungry by the time we walk back and it will be almost evening.” (Young woman in club refill, district hospital). Clients, therefore, tended to prefer the fast‐track model for ART refills to save time during the visits: “With fast‐track you are quickly served and go back home.” (Woman on routine HIV care, rural health centre). Clients enrolled in this model confirmed the convenience of fast‐track was confirmed by: “We just get here and collect our medication from the nurse […]” (Man in fast‐track, rural health centre). Fast‐track was also easy to integrate in their daily lives: “It saves time when you have work in the morning; for us farmers we need to be with our cattle and in our gardens, so we do not need to stay long at the clinic, and fast track can ensure that.” (Man on routine HIV care, district hospital). However, the implementation of fast‐track was challenging at understaffed clinics:

We are first treated there [at the outpatient department], then when our [patient] books get stamped we come here [to the pharmacy], then we sit on that bench. Sometimes, they start with the [other outpatient] patients as it will be extremely busy there, so even if you come very early [in the morning] sometimes you will be served around 2pm and the nurses are few […]. (Woman in fast‐track, rural health centre)

Concerns about waiting times were also mirrored in the healthcare providers’ observations about high workloads. Some facilities had only a few healthcare providers attending to large numbers of clients, which contributed to their feelings of being overwhelmed: “The workload is too much. We [do] everything.” (Healthcare provider, rural health centre). Some healthcare providers felt that group‐based refill models at times increased their workload instead of lessening it: “The work is too much, especially when we are supplying CARGs for medication.” (Healthcare provider, rural health centre).

## 4 DISCUSSION

We used a mixed‐method approach to assess the availability of differentiated ART delivery models in a rural district of Zimbabwe in 2019 and 2021. We explored the experiences of healthcare providers related to delivering HIV care and identified the issues that shaped preferences of PLHIV for some of the different models. Overall, 77% of all the facilities in the region offered at least one differentiated ART delivery model, with 50% offering only one option in addition to routine HIV care. Among the five differentiated ART delivery models recommended in Zimbabwe, CARG was the most offered model (50% of facilities) in the district, followed by fast‐track (31%) and family refill (23%). In contrast, club refill was only available at one district hospital (4%) and outreach was unavailable. This distribution reflects the early days of the rollout of differentiated ART delivery in rural Zimbabwe when staff training was only partial and client awareness of new models was still growing. Although only offered in eight facilities, fast‐track was reported as the preferred ART refill model across clients, regardless of whether they had enrolled on that model or not. Fast‐track was perceived as advantageous because it was the most likely to limit involuntary disclosure and the clinic visits were expected to be shorter. In contrast, for other clients who were not concerned by the risk of involuntary disclosure, CARGs were seen as a good option to reduce travel expenses.

Stigma is known to undermine adherence to ART and retention in care [26, 27, 28], thus concerns around privacy and disclosure need to be addressed while implementing any type of differentiated ART delivery. In our study, the confidentiality and privacy concerns highlighted the advantages of individual and facility‐based ART delivery models, such as fast‐track, over group‐based and community‐based models. It has been shown that HIV‐related stigma can deter PLHIV from accessing HIV services in their community because of the fear of disclosing the HIV status to neighbours or relatives [29, 30]. This was also documented in a study conducted in Zimbabwe, where stigma was the primary reason why men were not participating in CARGs [31]. The preference of PLHIV for facility‐based models in our study confirms recent findings from a study in urban Zimbabwe, where PLHIV preferred fast‐track over group‐based and community‐based models [32]. Conversely, facility‐based HIV care was considered stigmatizing in some urban Zambian and South African hospitals, where PLHIV were demarcated from others [33]. In our study, however, most of the facilities were small rural health centres, and there was no separation between PLHIV from other clients. Of note, disclosure of HIV status can be associated with increased retention in care [34, 35], and group‐based refill models can be a source of beneficial peer support [29, 36]. This has been shown by another study in Zimbabwe, where CARGs were valued for their additional peer support [37]. The variation in these findings underscores the complexity and need for well‐implemented client‐centred HIV care, balancing individual needs and concerns in different settings.

Travel costs were perceived as another barrier towards accessing HIV care at facilities. Long distances become problematic when clients have to walk for several hours to the facility instead of taking faster means of transport because of unavailability or higher costs. DSD, however, can decrease individual clinic visits and increase travel and cost savings to access HIV care [16, 29]. This was also shown in studies in Zimbabwe [37] and Malawi [12], where CARGs were associated with lower travel costs than routine HIV care. Multi‐month dispensing of ART could increase clients’ satisfaction by further reducing travel costs. Our study showed that a 3‐month supply was broadly implemented in Bikita District, and efforts to offer 6 months supply were ongoing. Unfortunately, we could not examine clients’ actual experiences of an outreach model in this study, as it was not implemented in the district due to the already limited resources, including the need for additional nurses, vehicles and drivers. Although the outreach model could benefit PLHIV living in hard‐to‐reach areas, confidentiality issues and stigma were feared by some clients and healthcare providers.

Our study identified waiting times as one of the main access challenges mentioned by clients. Yet, our quantitative data showed that clinic visit durations, from arrival upon departure, were generally short, especially for fast‐track members who had the shortest visits. This indicates a contradiction between main concern of clients (i.e. long waiting times), and the rather short duration of visit (including short waiting times) actually observed. This apparent contradiction reflects the recent reduction of crowding at facilities following the rapid increase in the number of HIV clinics as part of efforts to decentralize ART services in rural Zimbabwe [20, 38]. Visit times are also becoming shorter as most clients are stable and can obtain their ART refills without intensive clinical examination or counselling. In summary, the concern about long waiting times remains vivid despite recent improvements, which shows the importance of maintaining efficient services adapted to clients’ needs.

Healthcare providers felt overwhelmed by the workload caused by group‐based refill models, whereby members collecting drugs for their peers in addition to their clinical assessments contributed to the congestion of facilities where staff were already scarce. This underlines the challenges of implementing differentiated ART delivery models in practice, although DSD aims to relieve strain on health systems [3, 39]. Fewer visits over the year should balance out longer visit durations for CARG and family refill members if members go in turns to the facility, which was not observed in this study. We observed that the yearly number of visits was similar among clients enrolled in a group‐based model and those in routine HIV care. The relatively short time that the group models were available may explain why the decline in clinic visits had not yet fully materialized. It is also likely that clients participating in such models needed additional refills. This may be the case if clients are prescribed smaller refills due to insufficient ART supplies. Indeed, drug shortages are common at ART programs in resource‐limited countries, and impair supply, adherence and treatment outcomes [29, 40, 41, 42].

This study was limited by the difficulty in recruiting a representative sample of clients across target age groups, especially at smaller facilities with limited number of clients. However, the inclusion of smaller facilities contributed to a better understanding of real‐life implementation challenges, at places where access to HIV care is difficult. Most participating clients (80%) accessed ART refills through routine HIV care and a minority (20%) were enrolled in a differentiated ART delivery model. This may be explained by the fact that we did our study in the early days of DSD rollout in Zimbabwe, when healthcare providers were not yet offering DSD to all eligible clients. In addition, we observed that concerns about stigma may have discouraged some eligible clients from switching. Also, we recruited participants in care facilities, which may have favoured clients enrolled in facility‐based models or routine HIV care. Finally, it was challenging to distinguish fast‐track ART refill visits from full clinical review visits. This likely reflects the variable degree of fidelity in the early days of differentiated ART delivery model implementation. In this context, fast‐track refill visits could often also include some degree of clinical review. Furthermore, when only one or two healthcare providers dispensed HIV care at the time in a single room, clients on fast‐track and routine care were sometimes served in the same way. This shows the need for versatility in DSD implementation to suit the limited resources available in some rural settings, as well as the needs of the populations in care. We acknowledge that the clinic characteristics that influenced the choice of models implemented may also have influenced the perceptions of participants on the potential advantages or disadvantages of ART delivery options. Our study did not assess the fidelity of DSD implementation, which could give insights into how models are implemented in practice and tailored to local contexts, as well as the underlying decision‐making processes.

The strength of our study was the use of a mixed‐method approach to gain an in‐depth understanding of individual and sometimes contradictory perceptions of differentiated ART delivery in rural Zimbabwe. The qualitative arm of this study provided novel information about clients’ preferences and insights from healthcare providers related to the different ART delivery models available in the region, which should be generalizable to other settings in rural sub‐Saharan Africa. This topic would only be covered superficially in standardized questionnaires, especially the issues relating to confidentiality and disclosure. Furthermore, we were able to include all facilities in the rural district of Bikita, Zimbabwe, including a variety of care levels, from small health centres to larger hospitals, ensuring the representativeness of our results.

## 5 CONCLUSIONS

This study investigated the availability of differentiated ART delivery models in rural Zimbabwe and the preferences and challenges perceived or experienced by clients and healthcare providers. Confidentiality, travel costs and waiting times were perceived as the main concerns related to accessing ART, but perceptions and needs varied across participants. Fast‐track was often preferred for its privacy and short waiting times, while being easy to implement from the provider's perspective. In contrast, CARGs were subject to involuntary disclosure, but were appreciated for the reduced travel costs. Our study provides health authorities with timely information that should be considered to strengthen the delivery of ART in rural settings. Our data underline the importance of empowering clients to choose models of care meeting their needs. Health facilities should, therefore, provide a choice of models, if possible, including group models and fast‐track. Furthermore, offering longer refill periods could reduce the number of visits to the health facilities, while addressing the clients’ concerns for travel cost, confidentiality and waiting times, and the healthcare workers’ concerns for excessive workloads. In conclusion, we showed the importance of a flexible implementation of DSD to reflect the local context, resources and needs. Our study also shows the importance of implementation research to understand and guide the decision‐making processes underlying the DSD rollout in sub‐Saharan Africa, and to generate evidence on its acceptability, appropriateness and fidelity. In this context, the rigorous and standardized collection of data at regional and national levels will be essential to monitor and evaluate the effectiveness of differentiated HIV care.

## COMPETING INTERESTS

The authors declare that they have no competing interests.

## AUTHORS’ CONTRIBUTIONS

BC, JHVD, CK, FC, ME, AW and MB designed the study. TYN collected the data. BC, TYN and MLR analysed the data. BC, JHD, TYN, ME, AW and MB wrote the manuscript. All authors have read and approved the final manuscript.

## FUNDING

The research reported in this publication was supported by the U.S. National Institutes of Health's National Institute of Allergy and Infectious Diseases, the Eunice Kennedy Shriver National Institute of Child Health and Human Development, the National Cancer Institute, the National Institute of Mental Health, the National Institute on Drug Abuse, the National Heart, Lung, and Blood Institute, the National Institute on Alcohol Abuse and Alcoholism, the National Institute of Diabetes and Digestive and Kidney Diseases and the Fogarty International Center under Award Number U01AI069924, as well as by the Swiss National Science Foundation under Award Number 32FP30‐174281.

## DISCLAIMER

The content is solely the responsibility of the authors and does not necessarily represent the official views of the funders. ME was supported by special project funding (grant 189498) from the Swiss National Science Foundation.

## Data Availability

The data can be made available upon request according to our funder regulations.
